# Propoxur: MAK Value Documentation, addendum – Translation of the German version from 2024

**DOI:** 10.34865/mb11426e11_2ad

**Published:** 2026-06-30

**Authors:** Andrea Hartwig, MAK Commission

**Affiliations:** 1 Institute of Applied Biosciences, Department of Food Chemistry and Toxicology, Karlsruhe Institute of Technology (KIT) Adenauerring 20a, Building 50.41 76131 Karlsruhe Germany; 2 Permanent Senate Commission for the Investigation of Health Hazards of Chemical Compounds in the Work Area, Deutsche Forschungsgemeinschaft, Kennedyallee 40, 53175 Bonn, Germany. Further information: Permanent Senate Commission for the Investigation of Health Hazards of Chemical Compounds in the Work Area | DFG

**Keywords:** propoxur, insecticide, pesticide, toxicity, evaluation, acetylcholinesterase inhibitor

## Abstract

Propoxur [114-26-1] is used as an insecticide and acaricide. It is no longer approved in the European Union. The previous MAK Value documentation and addendum do not reflect the current data situation of the substance. The MAK Commissiondecided that a new evaluation is not of high priority. The MAK value and the other classifications are therefore suspended and the substance is listed in the Section II c of the List of MAK and BAT Values for substances no longer evaluated.

**Table:** 

**MAK value**	**see Section II c of the List of MAK and BAT Values**
**Peak limitation**	**–**
	
**Absorption through the skin**	**–**
**Sensitization**	**–**
**Carcinogenicity**	**–**
**Prenatal toxicity**	**–**
**Germ cell mutagenicity**	**–**
	
**BLW (2023)**	**reduction of the acetylcholinesterase activity in erythrocytes to 70% of the reference value^a)^**
	
Synonyms	2-isopropoxyphenyl *N*-methylcarbamate
Chemical name (IUPAC)	(2-propan-2-yloxyphenyl) *N*-methylcarbamate
CAS number	114-26-1
Molar mass	209.25 g/mol
Melting point	90.7 °C (IFA 2023)
Vapour pressure at 20 °C	1.29× 10^–5^ hPa (NCBI 2023)
log K_OW_	1.52 (IFA 2023)
Solubility at 20 °C	1.9 g/l water (IFA 2023)

^a)^ The BLW (biological guidance value) is derived as the ceiling value because of acute toxic effects.

This addendum was prepared because the previous evaluations no longer reflect the data currently available for the MAK value and for the designations and classifications of the substance. Propoxur is used as an insecticide and acaricide against many pests, especially sucking and biting insects and mites. In veterinary medicine, it is used as an agent against fleas in pets (AERU 2022). It is a carbamate insecticide that for a short time reversibly inhibits acetylcholinesterases. The biological guidance value (BLW) for acetylcholinesterase inhibitors (reduction of the acetylcholinesterase activity in erythrocytes to 70% of the reference value; Lewalter 1995; Weistenhöfer et al. 2024) therefore applies to propoxur. The BLW is derived as the ceiling value because of acute toxic effects. However, it was not investigated whether this is the most sensitive end point.

In 1979, a MAK value of 2 mg/m^3^ I (inhalable fraction) was derived. In 2001, propoxur was assigned to Peak Limitation Category II with an excursion factor of 8 (Greim 2002, available in German only; Henschler 1979, available in German only).

In the European Union, the use of propoxur as a pesticide is not permitted under Regulation (EC) 1107/2009 concerning the placing of plant protection products on the market (European Commission 2022; European Parliament and European Council 2009). Propoxur was approved for use in the Federal Republic of Germany from 1971 to 1999 and in the former German Democratic Republic until 1983 (BVL 2010), after which the substance was no longer authorized. Use as a flea repellent for pets (for example as a spray, shampoo or collar) is still approved in Germany (BfArM 2022).

The previous evaluations (MAK value documentation and addendum) do not reflect the currently available data. However, a re-evaluation of the substance is not a priority. Therefore, the MAK value, the peak limitation and the “H” designation have been withdrawn and propoxur has been allocated to Section II c of the List of MAK and BAT Values (DFG 2022). This section lists substances for which the previous MAK values, designations and classifications have been withdrawn and which are no longer being reviewed at present.
